# Toward a Digitally Informed Knitted Prosthetic Interface With Graded Stiffness to Enhance Comfort in Transtibial Amputees: Proof-of-Concept Case Study

**DOI:** 10.2196/91396

**Published:** 2026-06-26

**Authors:** Trevor Binedell, Pei Zhi Chia, Ying Yi Tan, Hong Yee Low

**Affiliations:** 1 Foot Care & Limb Design Centre, Tan Tock Seng Hospital, Singapore, Singapore; 2 Engineering Product Design Pillar, Singapore University of Technology and Design, 8 Somapah Road, Singapore, 487372, Singapore, 65 6499 4612

**Keywords:** prosthetic, health care, comfort, lines of nonextension, graded stiffness, multimaterial, knitting, textile, thermal reactive

## Abstract

**Background:**

Despite considerable advancements in prosthetic technology, a substantial proportion of lower limb amputees reduce or discontinue prosthesis use, with reported nonuse rates ranging from 12% to 53%. This reflects the multifactorial challenges associated with long-term prosthetic use, among which comfort and skin health are consistently identified as key determinants. More specifically, studies point toward nonbreathable silicone liners trapping heat and sweat, leading to skin and hygiene problems. These persistent limitations underscore the need for alternative interface materials that offer improved breathability, moisture management, and tunable mechanical properties.

**Objective:**

This study aimed to introduce Flexoknit, a transtibial prosthetic liner that integrates user-specific digital skin strain analysis with computer numerical control multimaterial knitting to create a mechanically tuned, breathable, and anatomically customized interface. Using digital biomechanical data as the primary design driver—rather than clinician heuristics alone—Flexoknit aims to determine the feasibility and performance of a skin strain–guided, computer numerical control–knitted prosthetic interface in terms of material function, clinical performance, and user experience.

**Methods:**

Flexoknit uses programmable multimaterial knitting, incorporating thermal-reactive yarns that stiffen when heated to create structural support zones, alongside spandex yarns that provide elastic compression and breathable zones. Uniaxial tensile tests showed that yarn and stitch combinations can generate distinct stiffness grades, with nearly order-of-magnitude differences. The spatial layout of these graded zones aligns high-stiffness regions with the lines of nonextension, and low-stiffness regions with areas of greater skin strain. With the new prosthetic interface, a series of controlled tests was conducted to compare performance against the participant’s existing prosthesis with a conventional silicone liner. User testing was organized into 3 domains (ie, mobility, suspension, and comfort) using standardized quantitative assessments and structured qualitative data collection.

**Results:**

User testing demonstrated a 22.5% improvement in total range of motion, a 37.5% reduction in interface mass, and improved thermal regulation in hot, humid environments compared to that of a conventional silicone liner. The user walked unaided and performed sit-to-stand movements, reporting positive comfort and usability feedback.

**Conclusions:**

This work establishes Flexoknit as a promising direction for future prosthetic development—one that integrates principles of biomechanics, textile engineering, and digital fabrication to create user-centered interface solutions. The findings suggest that digitally engineered knitted interfaces can provide a highly customizable, breathable, and compliant alternative to conventional silicone liners, particularly for lower-activity amputees or individuals prioritizing comfort and ease of use.

## Introduction

Despite considerable advancements in prosthetic technology, a substantial proportion of lower limb amputees reduce or discontinue prosthesis use, with reported nonuse rates ranging from 12% to 53% [1-3]. This reflects the multifactorial challenges associated with long-term prosthetic use, among which comfort and skin health are consistently identified as key determinants. Prosthetic comfort is central to user satisfaction and long-term adherence and strongly influences mobility and quality of life [4-8]. Interface-related discomfort—particularly heat, perspiration, and skin complications—has been repeatedly identified as a major contributor to reduced wear time and dissatisfaction among prosthesis users. Comfort is shaped by several factors, including socket design, interface materials, and suspension approaches [9], and each prosthetic socket is typically crafted through a manual, iterative process that aims to optimize fit [10]. For individuals with transtibial amputation, this precision is especially important because soft tissues must accommodate substantial loads and shear forces during everyday movement [11].

As the primary point of contact between the residual limb and the prosthetic socket, the interface plays a decisive role in user comfort. Dissatisfaction rates with this interface remain high: in a study of 78 lower limb amputees, only 43% reported being satisfied with prosthetic comfort [12]. Excessive sweating is one of the most frequently reported complaints among prosthesis users, affecting up to 70% of individuals with lower limb amputation and significantly impacting daily activities. Approximately half of users report discomfort related to heat and perspiration, highlighting the clinical significance of thermal burden within the socket-limb interface [13,14].

Silicone liners, while widely used to enhance suspension and load distribution, are inherently nonbreathable and act as thermal insulators, trapping sweat against the skin and exacerbating these issues. Among 83 amputees using silicone liners, 31% to 60% reported hygiene-related problems, including itching, perspiration, odors, and scarring [15]. Silicone’s insulating properties contribute to dermatological issues, such as heat rash, folliculitis, soreness, and odor [16], and users wearing liners have been shown to experience increased residual limb temperatures during exercise, whereas those without liners did not [17]. These issues are further intensified in hot and humid climates, where thermal load and moisture accumulation exacerbate discomfort [18]. Traditional strategies such as wearing socks under or over liners may improve fit but further increase insulation, adding to the thermal burden or reducing friction, which leads to a decreased coupling grip. These findings suggest that limitations in existing liner materials, particularly their inability to effectively regulate heat and moisture, represent a key modifiable contributor to prosthetic discomfort and reduced use.

These persistent limitations underscore the need for alternative interface materials that offer improved breathability, moisture management, and tunable mechanical properties. Textile-based solutions, particularly knitted structures, present a promising approach due to their inherent porosity, efficient moisture transport, and capacity for precise mechanical tuning. The intermeshing loop architecture of knitted fabrics can produce wide-ranging stiffness, stretch, and ventilation characteristics. Recent advances in textile engineering demonstrate that multimaterial knits can achieve spatially graded mechanical behavior through stitch topology [19], thermal-reactive yarns that form rigid 3D structures [20], and hybrid yarn systems that regulate functional responses [21]. However, existing prosthetic liners primarily rely on homogeneous elastomeric materials and do not exploit these textile capabilities. Early attempts to incorporate textile and knitted interfaces into prosthetic design have demonstrated several functional advantages but remain limited in scope. For example, fabric-covered or differentiated-stretch liner concepts—such as knitted tubes layered with silicone—have aimed to reduce pistoning by restricting distal elongation while preserving proximal compliance or modifying longitudinal elongation over pressure-sensitive areas, thereby improving suspension using the textile architecture [22]. Similarly, breathable textile liner designs using spacer fabrics and partial silicone coatings have shown improved ventilation, moisture transport, and cushioning, although these were largely reported in upper-limb applications and were individualized through manual trimming rather than digitally generated geometry [23]. In parallel, knitted compression systems for amputated limbs have demonstrated that stitch density, loop structure, and yarn selection can be used to tune compression and mechanical response; however, these designs were primarily intended for compression therapy or limb shaping rather than as load-bearing prosthetic interfaces [24,25]. More recently, digital workflows linking 3D scanning to knitting have demonstrated the feasibility of producing geometrically customized liners, but these approaches remain focused on geometric shape conformity and automated manufacturing [26].

Taken together, these studies establish that textile-based interfaces can improve breathability, cushioning, compression control, and even geometric customization. However, they do not yet demonstrate a prosthetic interface in which the regional mechanical properties of the material are prescribed based on subject-specific biomechanical data. In particular, prior work has not integrated digital skin strain mapping with multimaterial textile design to spatially align stiffness, compliance, and reinforcement with the mechanical demands of the residual limb.

A critical missing link in the pursuit of personalized prosthetic interface design is the integration of digital skin strain analysis into material architecture. Skin strain reflects the deformation of superficial tissues during movement, offering direct insight into where support, compliance, or protection is most needed. The concept of lines of nonextension (LoNEs)—regions of the skin that undergo minimal stretch during motion—first described by Iberall [27], has become increasingly adopted in the design of wearable devices. Aligning stiff materials with LoNEs and compliant materials in regions of high strain can minimize shear, reduce friction-related injuries, and enhance comfort.

Our previous work introduced a clinically accessible, low-cost digital workflow for mapping the surface strain field and identifying LoNEs on transtibial residual limbs using marker-based photogrammetry and open-source 3D reconstruction software [28]. This method quantifies maximum, minimum, and directional skin strains and reveals consistent patterns across individuals, including peak tensile strain over the patella, compression in the popliteal region, and anatomical yet individualized LoNE trajectories. These results extend prior studies using more complex imaging approaches [29], demonstrating that skin strain mapping provides actionable biomechanical data for prosthetic interface design.

The convergence of digital biomechanics and computer numerical control (CNC) multimaterial knitting presents an opportunity for medical device development. CNC knitting machines can precisely position yarns of different elasticity, stiffness, and thermal and moisture management properties, enabling the creation of functionally graded textiles that respond to the unique strain environment of each user. By integrating skin strain data with the knitting design process, stiff knitted structures can be strategically aligned along LoNE pathways to enhance suspension and reduce pistoning, while compliant, breathable knit zones can be placed in areas of high deformation to reduce friction, pressure, and heat accumulation.

Building on these innovations, this study introduces *Flexoknit*, a transtibial prosthetic liner that integrates user-specific digital skin strain analysis with CNC multimaterial knitting to create a mechanically tuned, breathable, and anatomically customized interface. Using digital biomechanical data as the primary design driver—rather than clinician heuristics alone—Flexoknit aims to determine the feasibility and performance of a skin strain–guided, CNC-knitted prosthetic interface in terms of material function, clinical performance, and user experience.

## Methods

### Study Design

This study used an experimental, mixed methods design to develop, characterize, and evaluate Flexoknit, a transtibial prosthetic interface created through CNC multimaterial knitting and informed by residual limb skin strain patterns. The study comprised three major phases: (1) *digital design and fabrication* of multimaterial knitted structures, including yarn selection, stitch programming, and interface construction; (2) *material and fabric characterization* involving thermal analysis, mechanical testing, and moisture management assessment of the knitted samples; and (3) *user testing* to evaluate mobility, suspension, thermal and moisture comfort, and overall user satisfaction compared with a conventional silicone liner.

This design enabled a comprehensive evaluation of both the engineered textile properties and the user-level functional response to a digitally informed prosthetic interface.

### Recruitment

Participants were recruited from a prosthetics clinic in Singapore using convenience sampling. Inclusion criteria included participants aged ≥21 years, unilateral transtibial amputation, regular prosthesis users, and the ability to ambulate independently with or without assistive devices. Exclusion criteria included active dermatological issues on the residual limb, acute musculoskeletal conditions, or medical conditions affecting ambulation.

One long-term transtibial prosthesis user (76-y-old man) completed the full user testing protocol, including mobility, suspension, thermal, moisture, and satisfaction assessments. An additional healthy participant (35-y-old man) performed the outdoor thermal test in hot and humid conditions to simulate typical Singapore climate exposure. The difference in participants between the indoor and outdoor tests was due to the age of the amputee participant. As the intention of the outdoor sweat and thermal test was to generate adequate sweat within a relatively short duration (15 min) by having a user who could exert themselves under the hot sun, the team opted for a healthy younger participant to perform this outdoor test instead.

### Materials and Fabrication

For the main body of the Flexoknit interface, 2 types of thermal-reactive yarns were used. TH1 consists of a 10/1 bicomponent polyester binder spun yarn supplied by Unitika, branded under the model ESPORAN. This yarn is constructed with a polyester core that has a melting point of 256 °C and a co-polyester sheath with a melting point of 110 °C. TH2 is made from a blend of polyester, polyurethane, and nylon, featuring a fusing point of 100 °C, supplied by Asahi Kasei. Three types of spandex yarns were used: ACY (300 D polyester DTY (96 F) and 100 D spandex); ZJS (210 D spandex and 2 ends of 75 D polyester, under the model number 2107575) from Zhejiang Kangjiesi New Material Technology Co., Ltd; and SPY, a low-stiffness elastic yarn from Shima Seiki called Shima Set Up Yarn.

**Figure 1.**
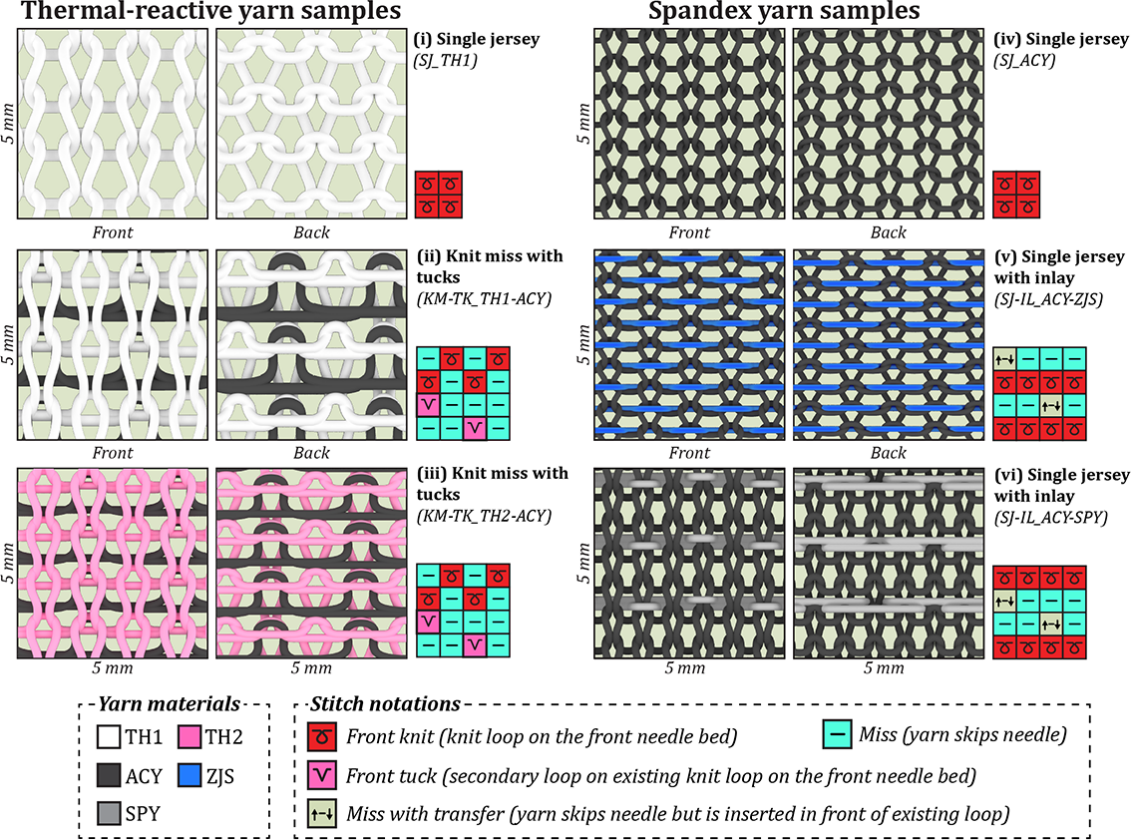
Stitch patterns and yarn combinations used in the knitted prosthetic interface. For each stitch pattern, a diagram of the front and back of a 5 mm square of fabric is shown, with a knit notation of the stitch pattern at the bottom right. There are three combinations that contain thermal-reactive yarn: (i) single jersey (yarn: TH1), (ii) knit-miss (TH1) with tucks (ACY), and (iii) knit-miss (TH2) with tucks (ACY). There are 3 combinations that do not contain thermal-reactive yarn: (iv) single jersey (ACY), (v) single jersey (ACY) with inlay (ZJS), and (vi) single jersey (ACY) with inlay (SPY).

The yarns were knitted into fabric on a Shima Seiki MACH2XS153-15L WHOLEGARMENT 15-gauge flat-bed weft knitting machine programmed using the SDS-ONE KnitPaint software. Three stitch patterns were used in the knitted prosthetic interface (see Figure 1 for diagrams of the stitch patterns). Single jersey (SJ), also known as plain jersey or stockinette stitch, is formed with only front knit stitches. SJ with inlay (SJ-IL) consists of 1 yarn to knit a SJ fabric, while a second yarn is woven in and out of the knitted loops of the first yarn. The second yarn forms approximately straight floats of yarn along the course of the fabric. Knit-miss (cross-miss) 1×1 with tucks (KM-TK) uses 1 yarn to knit the knit-miss 1×1 stitch pattern, where alternate needles knit and miss stitches, with the order of the knit and miss stitches reversing in every course. A second yarn tucks on the knit-miss 1×1 fabric at regular intervals.

More information on the fabrication of the prosthesis can be found in Multimedia Appendix 1.

### Material and Fabric Characterization

#### Yarn Materials

The 3 yarn materials that were used most extensively in the knitted prosthetic interface were characterized using a PerkinElmer DSC 6000 differential scanning calorimetry (DSC) machine. A sample of yarn weighing 7.5 to 8.6 mg was placed in a 50 µL aluminum pan and heated and cooled from 15 °C to 200 °C to 15 °C for 2 cycles at a rate of 10.00 °C per minute in a nitrogen atmosphere (20 mL per minute; see Table S1 in Multimedia Appendix 1 for more details).

#### Knitted Fabric

The uniaxial tensile properties of the knitted fabrics were characterized according to a modified standard referencing ISO (International Organization for Standardization) 13934‐1: 2014 (strip test) using an Instron 5943 with a 1.0 kN load cell (see Uniaxial tensile test sample preparation in Multimedia Appendix 1 for more details). The samples were first prepared by heating tubes of fabric on plywood blocks. As the knitted prosthetic interface is stretched in the course-wise (CW) direction when the interface is worn on the residual limb, the samples were made in 2 sizes: *regular* (tube circumference: 390 mm) and *narrow* (tube circumference: 286 mm or 26.7% narrower than regular tubes). The narrow tubes had to stretch to fit onto the plywood blocks. After heating, flat samples were cut out from the tubes to fit onto the tensile testing machine. Each sample size was 50 mm in width and 50 mm in gauge length, and each knit configuration was tested separately in 2 orientations: *wale wise (WW*) or 0° and *CW* or 90°. The strain rate used was 50 mm per minute across all samples. For each knit configuration, the force-strain curves were plotted, and the average breaking force, tensile strain at break, and elastic modulus were calculated.

For thermal and moisture characterization, 8 stitch patterns were tested using ACY yarn: interlock, mesh, pique lacoste (cross-tuck 1×1), purl (garter stitch), rib 1×1, SJ (plain jersey), single pique (cross-tuck interlock), and tuck 1×1. For each test, 3 samples were tested for each stitch pattern. Thermal resistance (K/W) and relative water vapor permeability (%) were measured using a fast skin model Permetest instrument (Sensora Instruments) in a manner similar to ISO 11092 [30]. Wicking rate was measured using a vertical wicking rate test [31,32], with the WW axis of the fabric aligned to the vertical axis. More information on the vertical wicking rate test can be found in Multimedia Appendix 1.

### Prosthetic Interface Fabrication

LoNEs are regions of the body that undergo negligible skin strain during movement [27]. Prior studies have suggested using LoNEs to guide material placement in wearable systems—including prosthetic sockets [33] and mechanical counterpressure suits [34]—by positioning stiff materials along low-strain areas and reserving compliant materials for regions of higher deformation. This strategy allows structural reinforcement without restricting natural skin mobility. Similar approaches have been applied in orthotic design using selectively reinforced 3D-printed elements [35].

**Figure 2.**
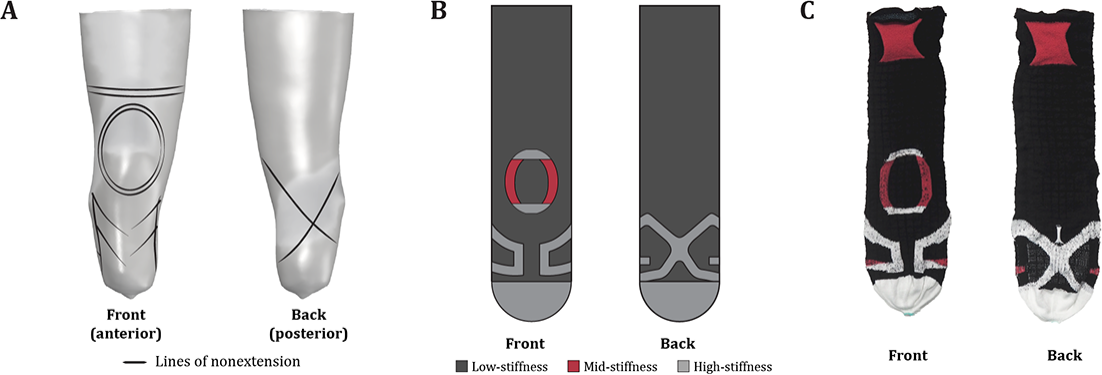
Design of the graded stiffness knitted prosthetic interface. (A) Diagram of lines of nonextension based on directional strain field from photographs of the user’s residual limb. (B) Diagram of high-, medium-, and low-stiffness zones on the prosthetic interface. (C) Photograph of the knitted prosthetic interface.

In this study, LoNEs informed the spatial layout of stiffness zones within the knitted prosthetic interface. The user’s residual limb was photographed in multiple poses, reconstructed as a 3D model, and analyzed to generate directional strain fields (Figure 2A; see [28] for full methodology). High-, medium-, and low-stiffness zones were then mapped based on these strain patterns (Figure 2B), forming the structural template for the knitted design. In this study, mapping was conducted under controlled, non–weight-bearing conditions, with the participant supported and not loading the residual limb. Skin deformation was captured between 2 standardized knee positions: full extension (0°) and flexion at approximately 60°, measured using a goniometer to ensure consistency.

For each posture, a circumferential video of the residual limb was recorded over approximately 90 seconds, from which a sequence of images (~300 frames) was extracted for 3D reconstruction. The strain field was computed by comparing the reconstructed limb geometry between the extended and flexed states, and the resulting directional strain data were used to identify LoNEs for interface design. A single mapping sequence was performed for the participant, and the derived strain field was used directly for design implementation.

This mapping approach captures kinematic skin strain behavior under controlled conditions and does not account for additional deformation under load-bearing scenarios. Accordingly, LoNEs are interpreted as functionally low-strain design pathways rather than fixed anatomical lines under dynamic loading.

High-stiffness zones were positioned directly over LoNE pathways, providing mechanical reinforcement without restricting mobility. These areas resist elongation under load, supporting suspension, minimizing pistoning, and redistributing pressure toward more tolerant regions of the residuum, such as the medial and lateral tibial surfaces and calf musculature [36]. A stiffened distal zone was added to improve load transfer at the socket-interface junction while reducing overall weight by confining rigid materials only to targeted regions.

Medium-stiffness zones were placed adjacent to the patella, where controlled flexibility is required to accommodate knee movement. This configuration provided directional stiffness in the vertical axis to prevent interface deformation and limit migration while preserving transverse flexibility for knee flexion.

Low-stiffness zones corresponded to regions experiencing high skin strain and were designed to stretch freely, supporting soft tissue deformation during movement. As these zones form most of the interface surface, using breathable, compliant yarns in these areas substantially improved comfort and thermal and moisture management. Integrated compression structures in both layers further improved mechanical coupling between limb and interface and helped manage shear.

**Figure 3.**
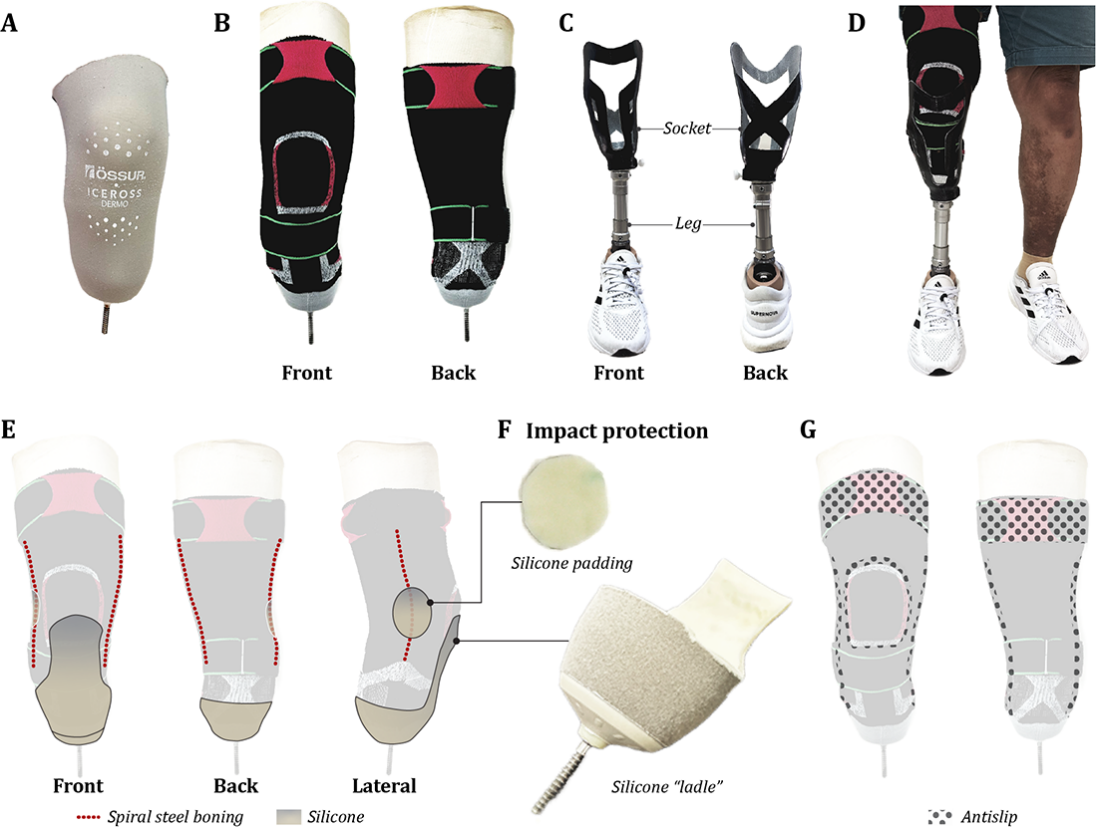
Prosthetic interface components. (A) Photograph of current Iceross silicone liner used by the user. (B) Photograph of experimental Flexoknit prosthetic interface. (C) Photograph of hard socket with artificial leg. (D) Photograph of complete Flexoknit prosthetic system. (E) Diagram showing locations of spiral steel boning and silicone padding for impact protection. (F) Photographs of silicone padding for impact protection. (G) Diagram showing the location of antislip.

The final Flexoknit interface was constructed as a double-layered knitted tube (Figure 3). The outer layer incorporated spatially graded stiffness elements through specific yarn and stitch combinations. High-stiffness zones were primarily knitted using the KM-TK_TH1-ACY configuration, while SJ_TH1 was used at the distal end to allow 3D shaping without compromising rigidity. Medium-stiffness zones used KM-TK_TH2-ACY, and lateral and medial regions included integrally knitted channels containing spiral steel boning. The boning provided vertical stiffness to reduce pistoning while preserving transverse flexibility for knee movement.

Low-stiffness regions were knitted with compliant spandex yarns (eg, SJ_ACY) to enable close limb conformity and accommodate local strain. Compression was strategically integrated: SJ-IL_ACY-SPY provided CW compression in the outer layer, while SJ-IL_ACY-ZJS applied surface-level compression in the inner layer, enhancing mechanical coupling and reducing shear.

Additional design features addressed impact protection, suspension, and adjustability. Cushioning pads were placed over vulnerable anatomical areas, including the fibular head, tibial crest, and distal stump. Suspension was facilitated by the thermoformed fit of the knitted tube, augmented by hook-and-loop straps, a patellar silicone ring, and antislip regions aligned with LoNEs. These elements improved security during ambulation without compromising donning ease or adaptability to limb volume fluctuations.

The knitted prosthetic interface was fabricated on a Shima Seiki MACH2XS153-15L WHOLEGARMENT 15-gauge flat-bed weft knitting machine, with all stitch programming executed in SDS-ONE APEX/KnitPaint. Thermal-reactive yarns (TH1 and TH2) were used to enable structural fixation after heat setting. During the user tests, the knitted prosthetic interface was inserted into a rigid laminated transtibial socket and aligned to ensure correct placement of stiffness zones.

### User Testing

Following fabrication of the Flexoknit prosthetic interface, a series of controlled tests was conducted to compare performance against the participant’s existing prosthesis with a conventional silicone liner. User testing was organized into 3 domains (ie, mobility, suspension, and comfort) using standardized quantitative assessments and structured qualitative data collection. A summary of the test protocol is provided in Table S2 in Multimedia Appendix 1.

#### Mobility Testing

##### Range of Motion

Range of motion (ROM) measurements were obtained using a standard goniometer. Joint angles were recorded for the hip, knee, and ankle with and without the prosthesis in place. ROM measurements followed the procedures described in Range of Motion in Multimedia Appendix 1.

##### Ten-Minute Walk Test

The participant was instructed to walk continuously for 10 minutes at a self-selected pace within a designated indoor gym space. Distance covered was recorded. Temperature readings were collected concurrently as described in Skin temperature measurements in Multimedia Appendix 1.

##### Ten-Meter Walk Test

Walking speed was assessed on a straight 10 meter walkway. To reduce participant burden, timings were obtained during the first 3 laps of the 10-Minute Walk test (10MinWT). The test was performed at a comfortable and safe pace.

##### Timed Up and Go

The Timed Up and Go (TUG) test followed a standardized procedure: starting from a seated position, the participant stood on cue, walked 3 meters, turned, returned to the chair, and sat down. Total completion time was recorded.

### Suspension Testing

A static elongation test was carried out. Two reference marks were applied along the proximal brim of the rigid socket—one at the highest lateral point and the other aligned with the patella. With the participant standing, the prosthetic limb was lifted while maintaining full knee extension. Vertical displacement between the original and displaced marks was measured to determine socket migration relative to the knitted interface. Displacement was measured manually using a ruler aligned to the reference markings, with a measurement resolution of 1 mm, and measurements were repeated across 3 trials for each prosthetic condition, with the mean value reported. The measurement error for vertical displacement using manual reference markings was estimated to be +1 or −1 mm based on repeated pilot measurements. Any measured variability across the 3 trials would also be reported as well.

Testing was performed with the knee maintained in full extension throughout to minimize variability due to joint position. Limb loading was standardized using the participant’s body weight during standing prior to limb lifting, followed by manual unloading of the prosthesis; however, no instrumented load measurement was used, and the applied force during limb lifting was not quantified.

This method represents a static assessment of suspension and does not account for dynamic loading conditions encountered during gait, including cyclic loading, shear forces, and stance-phase transitions. Given the manual measurement resolution and estimated error margin, small between-condition differences should be interpreted with caution. Supplementary feedback on suspension was collected through brief unstructured interviews.

### Comfort Testing

#### Mass Measurement

The total mass of each prosthetic configuration was recorded using a digital scale (Model 103272; Redman). Socket mass was measured separately, and interface mass was calculated as the difference between total system mass and socket mass. The same procedure was used for both the silicone liner system and the Flexoknit system.

#### Thermal Comfort

Thermal measurements were collected during the indoor 10MinWT. The indoor environment was measured at 23 °C, and the participant was dressed in a T-shirt and shorts on the day of the test. A ProtoCentral MAX30205 wearable body thermometer was secured to the center of the calf muscle belly. This location was selected due to the muscle’s role in generating heat during physical activity [37,38]. Continuous skin temperature data were recorded throughout the 10-minute walking period for both liner conditions.

As the 10MinWT was conducted indoors in an air-conditioned clinic, a secondary experiment was designed to evaluate thermal performance in a warm, humid outdoor environment, reflective of typical daytime conditions in Singapore (31 °C‐33 °C) [39] and reflective of a more accurate simulation of prolonged heat exposure. A healthy participant (35-y-old man), who was dressed in a T-shirt and shorts, wore customized versions of the silicone and knitted interfaces with dual openings for ease of donning. Each test included a resting period (5‐15 min or until temperature stabilization) followed by 15 minutes of continuous walking on a partially sun-exposed track. Temperature was recorded using the same protocol as the indoor testing.

#### Moisture Comfort

Moisture comfort was assessed through verbal feedback and postactivity inspection of the prosthetic interface. Examinations focused on the presence of sweat accumulation and tactile dampness. Observations were documented immediately following each walking trial for both interface types.

### User Experience and Satisfaction

#### Standardized Questionnaires

Two validated user-reported outcome measures were administered: (1) Socket Comfort Score (SCS) [40] and (2) Quebec User Evaluation of Satisfaction with Assistive Technology (QUEST 2.0).

The participant provided independent ratings for the conventional liner and Flexoknit conditions.

#### Qualitative Interviews

Unstructured interviews were conducted to gather additional feedback related to perceived comfort, usability, adjustability, and overall experience with each interface. Real-time observations of interface behavior during walking and donning were also recorded.

### Data Analysis

Material properties (DSC peaks, breaking force, strain at break, modulus, thermal resistance, vapor permeability, and wicking rate) were analyzed. Tensile results were plotted as force-strain curves. Mobility and suspension outcomes were compared between the Flexoknit interface and the participant’s silicone liner. Temperature data were analyzed as continuous time series profiles. Moisture comfort and qualitative feedback were thematically summarized. Questionnaire scores (SCS and QUEST 2.0) were tabulated and compared across configurations.

This study comprised a material characterization phase and a single-user proof-of-concept evaluation. Owing to the single participant design of the user testing component (n=1), no inferential statistical analysis was performed for the clinical, functional, thermal, or satisfaction outcomes. Normality testing and hypothesis testing were not appropriate for single participant data. User testing results are therefore presented descriptively as raw values and percentage differences between the experimental and conventional prosthetic conditions.

For specific outcome measures, ROM, gait, mass, thermal comfort, and SCS data are reported as observed values, while QUEST 2.0 results are summarized using median scores. Suspension testing using the static elongation method is reported descriptively, with mean displacement values presented for each condition.

Material and fabric characterization data were also analyzed descriptively. Thermal and moisture management results are presented as mean values, as presented in Table 1, and mechanical behavior is presented using observed ranges and representative force-strain responses across yarn-stitch combinations. As this study was designed as a proof-of-concept investigation, no inferential comparisons were performed.

**Table 1.** Thermal and moisture comfort results.

Stitch pattern	Thermal resistance		Water vapor permeability		Vertical wicking rate	
	Mean (m²·K/W)	Rank	Mean (%)	Rank	Mean (mm/s)	Rank
Interlock	40.20	3	35.97	6	0.1437	7
Mesh	40.67	4	45.13	2	0.1971	3
Pique lacoste	41.63	5	41.87	5	0.2115	2
Purl	35.68	2	42.33	4	0.1439	6
Rib 1×1	58.97	8	34.27	7	0.0843	8
Single jersey	31.95	1	47.47	1	0.1964	4
Single pique	48.23	7	30.60	8	0.1755	5
Tuck 1×1	42.48	6	42.60	3	0.2778	1

### Ethical Considerations

This study was approved by the Institutional Review Board of the Singapore University of Technology and Design (HBR-22‐00507). All participants provided written informed consent prior to participation. Participants were informed of the study objectives, procedures, potential risks, and benefits, and were free to withdraw from the study at any time without penalty. All data were deidentified prior to analysis and reporting to protect participant privacy and confidentiality. Only the study investigators had access to identifiable participant information, which was stored securely in accordance with institutional data protection requirements. Participants did not receive financial compensation for participation in this study.

## Results

Results are presented according to the 4 methodological domains: material and fabric characterization, user testing results (mobility, suspension, and comfort), user experience, and user satisfaction.

### Participant Characteristics

One transtibial prosthesis user (76-y-old man, height 173 cm, weight 78 kg, and 15 y post amputation due to diabetes) completed the full mobility, suspension, comfort, and user experience testing. The participant’s conventional prosthesis consisted of a 6 mm silicone locking liner, laminated composite socket, pylon components with a pin lock suspension system, and a 1C30 Trias foot, worn for an average of 9 hours daily.

### Material and Fabric Performance

#### Thermal Characteristics (DSC Results)

**Figure 4.**
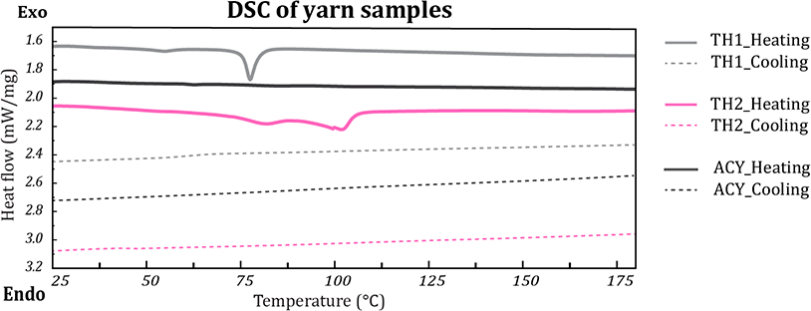
Differential scanning calorimetry (DSC) thermograms for the 3 types of yarns: TH1, TH2, and ACY. The solid lines represent heating, while the dashed lines represent cooling.

DSC revealed distinct thermal behaviors among the yarns (Figure 4). TH1 exhibited a single melting peak at approximately 75 °C, while TH2 showed 2 broad melting transitions at approximately 75 °C and 110 °C. The ACY spandex yarn demonstrated no significant thermal transitions within the tested range.

#### Mechanical Behavior of Knitted Fabrics

**Figure 5.**
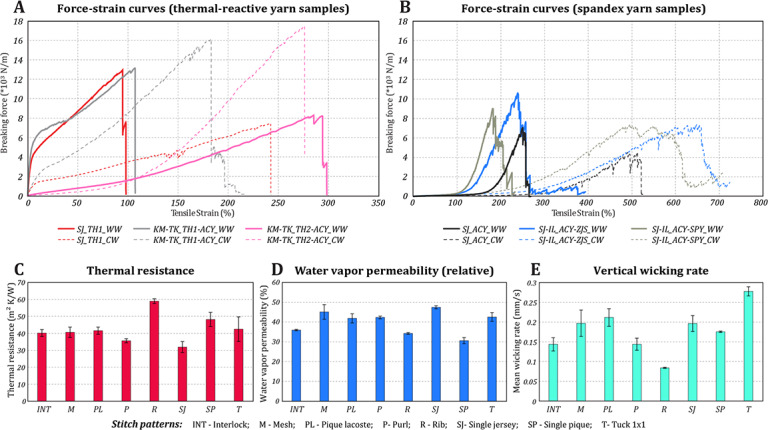
Material characterization results. (A, B) Uniaxial tensile tests for 6 stitch pattern and yarn combinations (regular-width samples only). (A) Force-strain curves for samples containing thermal-reactive yarn and (B) force-strain curves for samples that do not contain thermal-reactive yarn. (C-E) Thermal and moisture comfort results for 8 stitch patterns knitted with ACY yarn. (C) Thermal resistance, (D) relative water vapor permeability, and (E) vertical wicking rate.

Uniaxial tensile testing demonstrated clear differentiation in mechanical behavior across the yarn-stitch combinations (Figure 5). Fabrics incorporating thermal-reactive yarns exhibited higher breaking forces and lower tensile strains at break compared with those knitted solely from spandex-based yarns. Analysis of elastic modulus values showed that the knitted samples consistently fell into 3 stiffness categories:

- Low stiffness (38‐288 N/m): SJ_ACY, SJ-IL_ACY-SPY, SJ-IL_ACY-ZJS
- Medium stiffness (1224‐2978 N/m): KM-TK_TH2-ACY
- High stiffness (12,886‐110,337 N/m): SJ_TH1, KM-TK_TH1-ACY

The inclusion of thermal-reactive yarns resulted in substantially higher stiffness and strength at break. For example, KM-TK_TH1-ACY exhibited an elastic modulus nearly an order of magnitude greater than KM-TK_TH2-ACY, despite both samples using the same stitch architecture. KM-TK_TH1-ACY showed a more brittle stress-strain response, whereas KM-TK_TH2-ACY demonstrated greater elasticity, with pronounced anisotropy characterized by higher stiffness in the CW direction than in the WW direction.

To evaluate the effect of prestretching during heat setting, both regular and narrow samples were tested. Narrow samples, which replicated the stretch applied when forming the knitted interface over a mold, generally exhibited increased stiffness and breaking force in the CW direction, along with reduced tensile strain at break. In the WW direction, narrow samples typically showed decreased breaking force, increased strain at break, and reduced stiffness. An exception was SJ_TH1, which demonstrated increased stiffness in both directions when tested in the narrow state. Across all combinations, narrow samples showed more homogeneous mechanical behavior between axes, indicating reduced anisotropy after prestretching. No systematic differences were observed between front and back sample orientations.

In addition, the force-strain curves in Figures 5A and 5B revealed the distinct failure modes of the different knit samples. Upon testing the samples to their breaking point, the thermal-reactive yarn samples exhibited brittle failure with a sudden force drop, possibly due to the presence of hardened thermoplastic (TH1 and TH2) in the knit. The failure mode is akin to a cleavage in the middle of the sample’s testing area. On the other hand, the spandex yarn samples exhibited ductile tensile failure with significant “noise” upon breaking. The tested samples showed necking before failure, followed by several consecutive instances of columns or rows of elastic yarn loops breaking and unraveling upon failure. This behavior was reflected as several step-down peaks after the sample reached its breaking force.

#### Thermal and Moisture Management Performance

Eight stitch patterns were evaluated for thermal resistance, water vapor permeability, and wicking rate (Table 1). SJ demonstrated the lowest thermal resistance and highest vapor permeability, with a mid-range wicking rate, ranking highest overall for thermal and moisture management performance.

### User Testing

#### Mobility Outcomes

##### Range of Motion

ROM testing showed measurable differences across prosthetic configurations. Using the knitted interface alone, maximum knee extension remained unchanged, while maximum flexion decreased by 10°. When used with the LoNE-aligned rigid socket, total knee ROM was 98°, compared with 80° when using the participant’s current prosthesis (silicone liner and hard socket). In both conditions, terminal knee extension was limited to 20° of flexion (ie, a 20° extension deficit). Total ROM was calculated as the difference between peak flexion and terminal extension, and the 18° increase in ROM for the knitted interface and LoNE-aligned rigid socket is therefore attributed to increased flexion capability rather than a change in extension (Table 2).

**Table 2.** Range of motion (ROM) measurements of the various prosthetic configurations.

Prosthesis type	Extension (°)	Flexion (°)	Total ROM (°)
None (bare skin)	0	130	130
Knitted prosthetic interface	0	120	120
Current prosthesis	20	100	80
Experimental prosthesis	20	118	98

##### Gait Performance

During the 10MinWT, the participant covered 10.5% less distance with the experimental prosthesis compared with the conventional system. In the 10-Meter Walk Test (10mWT), walking time increased by 8.3% when using the knitted interface. The TUG test duration increased by 1.7 seconds. The participant ambulated independently during all tests.

### Suspension Outcomes

Socket migration measured using the static elongation test showed a mean displacement of 4 mm (+1 or −1 mm) with the experimental system and 3 mm (+1 or −1 mm) with the conventional system. Given that the observed between-condition difference (1 mm) lies within the estimated measurement error of the manual marking method (+1 or −1 mm), this finding should be interpreted with caution. During indoor walking, the participant reported a gradual loosening sensation and sinking at the distal tibia when using the experimental prosthesis, despite the small measured difference in static displacement. These observations were recorded during the structured suspension assessments.

### Comfort Outcomes

#### Mass

The experimental prosthesis weighed 26.1% less than the participant’s current prosthesis. The knitted interface alone was 37.5% lighter than the silicone liner.

#### Thermal Comfort

In the indoor air-conditioned setting (23 °C), the user’s resting posterior limb skin temperature increased by 0.89 °C with the knitted interface and 0.18 °C with the silicone liner. During the 10MinWT, the skin temperature increased by 0.82 °C (knitted) and 0.62 °C (silicone).

However, in the warmer outdoor setting (34 °C), the nonamputee’s resting posterior limb skin temperature increased by 0.21 °C (knitted) and 0.71 °C (silicone). During the 10MWT, the skin temperatures rose by 1.95 °C with the knitted interface and 2.88 °C with the silicone liner.

#### Moisture Comfort

No sweat accumulation was observed in indoor trials. In outdoor trials, visible perspiration accumulated beneath the silicone liner, and the participant reported a sweaty sensation. The knitted interface left the skin dry on doffing, although the fabric itself was damp. A sharp drop in skin temperature after removing the silicone liner was observed; no similar drop occurred with the knitted interface.

### User Experience and Satisfaction

#### Socket Comfort Score

The participant rated the knitted interface 7/10 and the current prosthesis 9/10.

#### QUEST 2.0 Satisfaction

**Figure 6.**
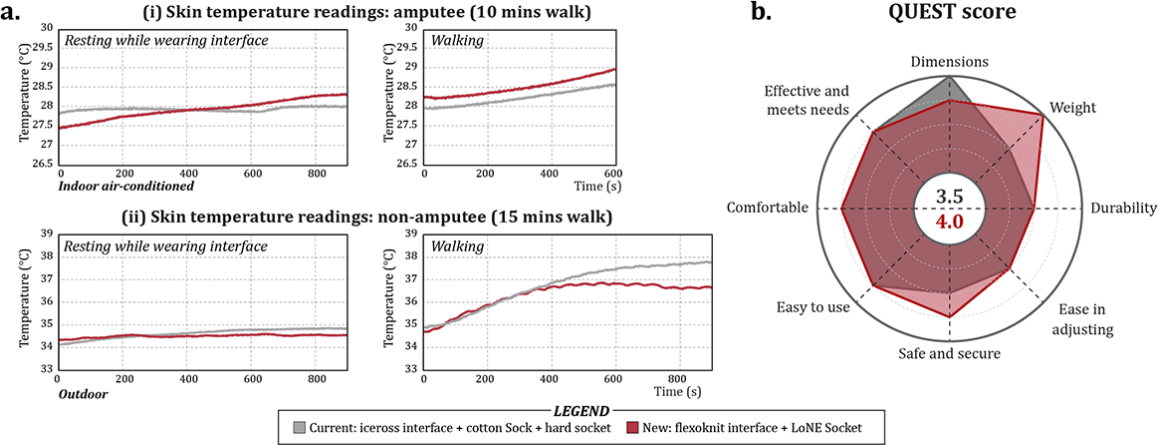
(A) Skin temperature measurements when resting while wearing the prosthetic interface and walking while wearing the prosthetic interface and socket for (i) the amputee and (ii) the nonamputee users. (B) Quebec User Evaluation of Satisfaction with Assistive Technology (QUEST) score showing the user-reported outcomes for the current and experimental prostheses. The numbers in the center of the spider diagram show the median scores for the current (black) and experimental (red) prostheses. A score of 1 represents “not satisfied at all,” and a score of 5 represents “very satisfied.” LoNE: line of nonextension.

The knitted prosthesis received a higher median satisfaction score (4.0) than the conventional prosthesis (3.5; Figure 6B). The IQR of the knitted prosthesis’s score was 0.5, while the conventional prosthesis was 1.0, demonstrating that there was less variation in the participant’s responses for the knitted prosthesis than the conventional one.

#### Qualitative Feedback

Qualitative feedback from interviews and real-time observation provided additional insight into the participant’s experience using both prosthetic systems. When using the Flexoknit interface, the participant consistently remarked on the noticeably reduced weight of the device, describing it as feeling lighter and more compliant during walking. The knitted structure was perceived as “springy,” and the participant noted a softer underfoot feel compared with his usual prosthesis. In indoor trials, the participant reported that the knitted interface felt warmer around the limb, whereas in outdoor trials, the nonamputee participant described the knitted interface as feeling cooler and less sweaty than the silicone liner. The textile appearance of the knitted interface was preferred, with the participant expressing positive reactions to its aesthetic qualities.

During donning, the participant reported that the hook-and-loop suspension straps added steps to the usual routine and occasionally caught on the surface of the knitted fabric, although no structural damage was observed. While walking with the experimental prosthesis, the participant described a gradual sinking sensation at the distal tibia, corresponding to the mild static elongation observed during suspension testing. After returning to the conventional prosthesis following the experimental trials, the participant described the device as feeling comparatively “hard” during stance and walking, indicating a perceptible difference in interface compliance between the 2 systems.

## Discussion

### Principal Findings

This proof-of-concept study demonstrates the feasibility of a digitally informed, multimaterial knitted prosthetic interface for transtibial users while also highlighting the biomechanical trade-offs introduced by increased interface compliance. By combining residual limb skin strain analysis with CNC multimaterial knitting, we created Flexoknit—a double-layered, graded stiffness interface integrated with a LoNE-informed rigid socket. Material characterization showed that targeted yarn-stitch combinations can generate 3 distinct stiffness tiers spanning nearly an order of magnitude in elastic modulus. When implemented in a transtibial system, this graded design reduced interface mass relative to a conventional silicone liner, preserved or improved knee ROM, and showed context-dependent thermal and moisture advantages, particularly in hot outdoor conditions. However, suspension-related challenges, including the participant’s reported distal “sinking” sensation, and the reduced distance walked during the 10MinWT indicate that improvements in ROM and comfort-related properties do not necessarily translate into better gait performance or interface stability. Despite the suspension-related challenges and donning complexity, user-reported satisfaction scores and qualitative feedback indicate that the concept is acceptable and potentially attractive for lower-activity community amputees. Taken together, these findings suggest that increased interface flexibility may have facilitated greater knee excursion during isolated ROM testing, while simultaneously reducing stance-phase stability during walking through less consistent limb-socket coupling. These findings therefore support feasibility but also highlight the need for further refinement of load transfer and suspension security before broader clinical application.

### Design of Multimaterial Graded Stiffness Knitted Fabric

The material characterization phase confirms that both yarn composition and stitch architecture can be systematically tuned to control mechanical behavior. Thermal-reactive yarns (TH1 and TH2) exhibited clear melting transitions and, once heat-set, produced knitted structures with substantially higher stiffness and strength than fabrics knitted solely from spandex yarns, consistent with prior work on thermal-reactive knits [20]. Uniaxial tensile testing showed that KM-TK_TH1-ACY and SJ_TH1 formed the high-stiffness group, KM-TK_TH2-ACY formed the medium-stiffness group, and SJ spandex configurations formed the low-stiffness group. The separation between these categories—38‐288 N/m; 1224‐2978 N/m and 12,886‐110,337 N/m, respectively—provided a clear design palette for allocating support and compliance within the interface.

Prestretching the knitted tubes prior to heat setting, a process that mimics forming the fabric over a limb mold, further modified the mechanical response. Narrow (stretched) samples typically showed increased stiffness and breaking force in the CW direction and more homogeneous behavior between WW and CW axes, suggesting reduced anisotropy once the fabric is tensioned to its in-use configuration. This behavior is advantageous for prosthetic applications, where a predictable, direction-independent response can improve suspension and load transfer.

Thermal and moisture management testing indicated that stitch patterns can be used to optimize comfort-related properties independently of stiffness. Among the patterns assessed, SJ achieved the best overall balance of low thermal resistance, high water vapor permeability, and adequate vertical wicking, supporting its use as the dominant structure in low-stiffness, high-contact zones. Collectively, these findings show that multimaterial CNC knitting can deliver a controlled spectrum of mechanical, thermal, and moisture management properties within a single seamless interface.

### LoNE-Guided Reinforcement Design

The LoNE-guided, digitally manufactured construct illustrates a shift from uniform material liners toward spatially tailored interfaces in which stiffness, compression, and breathability are all driven by patient-specific biomechanical data. LoNE-guided reinforcement may improve suspension by placing stiffer load-bearing elements along relatively low-strain pathways, where they can resist migration with less disruption to natural tissue motion. However, LoNEs are not assumed to remain perfectly fixed under load-bearing conditions and should instead be interpreted as functionally stable design zones. Nevertheless, the present findings suggest that anatomical alignment of stiffness zones alone is insufficient; effective suspension depends not only on alignment with LoNE pathways but also on the absolute magnitude and spatial distribution of interface stiffness required to maintain stable load transfer during dynamic gait.

### Interpretation of User Testing and Clinical Implications

In functional testing with the participant, the Flexoknit system preserved maximum knee extension and increased overall knee flexion when compared with the conventional prosthesis, yielding a 22.5% improvement in total ROM. This suggests that aligning stiff zones with LoNEs and reserving compliant structures for high-strain regions may permit greater sagittal-plane excursion at the knee without immediately compromising basic structural support. However, the increase in ROM should not be interpreted as a direct proxy for improved functional mobility, as gait performance during the 10MinWT, 10mWT, and TUG was modestly worse with the experimental system. Despite the 26.1% reduction in prosthesis mass, the participant walked more slowly with the experimental system. This suggests that prosthetic weight alone was not the dominant determinant of performance in this case. From a biomechanical perspective, walking efficiency depends not only on limb mass but also on the quality of load transfer, proprioceptive consistency, and the user’s confidence during the stance phase. It is therefore plausible that the benefits of reduced mass were offset by less stable mechanical coupling at the limb-socket interface. Importantly, the participant was able to ambulate independently and perform functional tasks such as sit-to-stand, indicating that the experimental system met minimum requirements for community mobility.

The participant’s report of a gradual distal tibial “sinking” sensation is biomechanically significant, suggesting incomplete load transfer coupling between the residual limb, knitted interface, and rigid socket. In an optimally coupled system, axial and shear forces are distributed proximally and circumferentially to maintain stable limb positioning. In this case, the increased compliance of the knitted interface likely permitted localized deformation—particularly distally—reducing the efficiency of axial load transfer. This may explain the perceived distal settling despite only a 1 mm difference in static elongation, highlighting that small displacements can be clinically meaningful when repeatedly experienced at sensitive anatomical sites.

Although the study was not designed to isolate the precise structural cause, this behavior likely reflects a combination of compliant material response and insufficient longitudinal reinforcement in low-stiffness regions. While these compliant zones were intended to accommodate soft tissue and enhance comfort, they may have allowed excessive vertical deformation under load. Future iterations should therefore refine not only overall stiffness but also the spatial distribution of reinforcement to improve suspension without compromising comfort.

This phenomenon also has implications for gait stability. While compliance can enhance pressure distribution, excessive deformation may introduce micromovements that reduce proprioceptive consistency and perceived security during stance. This may contribute to a more cautious gait pattern, reduced efficiency, and slower functional performance, even in the absence of pain. The participant’s slower walking speed and longer TUG time are therefore likely influenced not only by device unfamiliarity but also by altered mechanical coupling at the interface, potentially reducing confidence during weight acceptance and forward progression.

The graded stiffness design also achieved a substantial reduction in mass, with the experimental prosthesis approximately one-quarter lighter overall and the knitted interface over one-third lighter than the silicone liner. The participant’s perception of the prosthesis as “lighter” and “springy” aligns with these findings and may explain the increased knee flexion observed during ROM testing. However, this reduction in mass did not translate to faster gait. The perceived “springiness” likely reflects increased compliance, whereby some mechanical energy during stance was absorbed within the interface rather than efficiently transferred to forward progression. While this may enhance comfort, it may also reduce the stability required for confident movement. These findings suggest that reductions in prosthetic weight should be considered alongside interface stability, as improvements in one may be offset by compromises in the other.

Thermal and moisture outcomes were environment dependent. In the air-conditioned clinic, the knitted interface produced slightly greater temperature rises than the silicone liner, suggesting increased insulation when ambient temperatures are low. In contrast, in a hot outdoor setting representative of tropical climates, the knitted interface limited skin temperature rise and left the skin dry after walking, while the silicone liner trapped sweat and was perceived as hot and sticky. These findings imply that an open, wicking knitted structure may offer particular advantages in warm, humid environments, where dermatological complications and heat intolerance are common.

Despite generally adequate suspension, the static elongation test and user reports indicated minor distal “sinking” with the experimental system. This finding likely reflects the combined mechanical behavior of the compliant knitted interface, the stiffness zoning strategy, and the structural response of the LoNE-informed rigid socket under dynamic load. Rather than representing gross pistoning alone, the sensation may indicate localized compression and delayed load sharing across the limb-liner-socket complex, particularly during repeated stance loading. Clinically, this is relevant because even subtle distal displacement can alter pressure concentration, disturb the user’s perception of stability, and reduce confidence during walking. This observation may also help explain the slower walking speed and longer TUG duration despite the reduced prosthetic mass. Even subtle distal settling or micromovement at the interface can alter how ground reaction forces are transmitted through the prosthesis and may reduce the user’s confidence during loading response and mid-stance. In response, the user may adopt a more cautious gait strategy, with slower progression and more conservative transfer during transitional movements such as turning and sit-to-stand. This likely reflects the combined behavior of the compliant interface and the LoNE-informed rigid socket, including possible flexing of the latter under load.

The lower SCS for Flexoknit compared with the user’s own device appears to be driven by this sensation of gradual loosening rather than by gross discomfort or pain. This interpretation is also consistent with the higher overall QUEST score and positive qualitative feedback regarding appearance, weight, and perceived compliance, suggesting that user acceptance of the concept remained favorable despite the mechanical shortcoming. Taken together, these findings suggest that interface compliance must be carefully balanced: sufficient to accommodate tissue motion and improve comfort, but not so great that it compromises distal support or mechanical coupling.

Future iterations should therefore investigate increasing distal and longitudinal stiffness, refining the suspension mechanism, and optimizing outer socket rigidity to reduce perceptible settling during gait. Nonetheless, the higher overall QUEST score and the participant’s preference for the device’s appearance and perceived security suggest that, with refinements to the outer socket stiffness and suspension strategy, user comfort could be further improved.

### Conclusions

This study presents a proof-of-concept transtibial prosthetic interface—Flexoknit—engineered through a multimaterial, graded stiffness knitting approach informed by LoNEs. By combining thermal-reactive yarns, spandex-based elastic yarns, and spatially targeted stitch architectures, the knitted interface achieved distinct zones of mechanical behavior that were aligned with the user’s residual limb strain patterns and functional requirements. The fabrication process demonstrated that CNC knitting can reliably integrate structural reinforcement, compliance, compression, and breathability within a single seamless textile construct tailored to an amputee’s limb geometry.

Material testing confirmed that the yarn-stitch combinations produced the intended stiffness gradients, while functional testing showed that the interface supported safe ambulation and preserved or enhanced joint mobility when used with a LoNE-aligned rigid socket. Improvements in thermal and moisture regulation, particularly in warm outdoor conditions, and a substantial reduction in overall prosthesis weight further demonstrated the potential benefits of integrating knitted structures into prosthetic design. User feedback highlighted the comfort, lightness, and aesthetic appeal of the device, reinforcing the feasibility of this approach for improving user experience.

As an early prototype, some usability challenges remain, including potentially inadequate prosthesis stability under load and the need for more intuitive suspension and donning mechanisms. Nonetheless, the findings suggest that digitally engineered knitted interfaces can provide a highly customizable, breathable, and compliant alternative to conventional silicone liners, particularly for lower-activity amputees or individuals prioritizing comfort and ease of use.

Overall, this work establishes Flexoknit as a promising direction for future prosthetic development—one that integrates principles of biomechanics, textile engineering, and digital fabrication to create user-centered interface solutions. Further research is warranted to refine structural performance, evaluate long-term durability, and validate the design across diverse user groups and real-world conditions.

### Limitations and Future Work

This study has several limitations that should be considered when interpreting the findings. First, the prosthetic evaluation was conducted with a single transtibial amputee user, and thermal testing in a hot outdoor environment involved only one additional healthy participant. The results therefore reflect an in-depth case study rather than population-level evidence and may not generalize to users with different limb geometries, activity levels, or comorbidities. Second, the familiarization period with the Flexoknit system was short, so the gait and comfort outcomes likely include early adaptation effects. Longer-term use may alter both objective performance and subjective preferences. Third, the prototype system combined a novel knitted interface with a bespoke LoNE-informed rigid socket that has not yet been fully optimized. The biomechanical interpretation of distal “sinking” is based on user reports, static elongation data, and observational findings rather than instrumented assessment of in-socket motion or pressure distribution. In addition, while material and bench-top testing were extensive, real-world durability, laundering behavior, and the long-term skin response to continuous fabric–skin contact were not assessed. These aspects are critical for translation to everyday clinical practice.

Future work should therefore focus on multiparticipant studies that include a broader range of ages, activity classifications, and residual limb shapes, with follow-up over weeks to months. Such studies should incorporate more detailed biomechanical outcomes (eg, 3D gait analysis, interface pressure, and shear mapping) and dermatological assessments to evaluate skin integrity over time. On the design side, iterative optimization of the rigid socket stiffness, strap layout, and donning sequence is needed to reduce distal sinking and simplify daily use. Finally, further development of the digital workflow—from automated LoNE extraction to direct translation into knit patterns—along with systematic durability and wash-cycle testing, will be essential to move Flexoknit from proof-of-concept to a clinically deployable, manufacturable system.

## Supplementary material

10.2196/91396 Multimedia Appendix 1 Supplementary methods and materials used in this paper.

## Abbreviations

10MinWT: 10-Minute Walk Test
10mWT: 10-Meter Walk Test
CNC: computer numerical control
CW: course-wise
DSC: differential scanning calorimetry
ISO: International Organization for Standardization
KM-TK: knit-miss (cross-miss) 1×1 with tucks
LoNE: lines of nonextension
QUEST: Quebec User Evaluation of Satisfaction with Assistive Technology
ROM: range of motion
SCS: Socket Comfort Score
SI-IL: Single jersey with inlay
SJ: single jersey
TUG: Timed Up and Go
WW: wale-wise
