# Supplementary material for: Toward a Digitally Informed Knitted Prosthetic Interface With Graded Stiffness to Enhance Comfort in Transtibial Amputees: Proof-of-Concept Case Study
Source: JMIR Rehabil Assist Technol. 2026 Jun 26;13:e91396. doi: 10.2196/91396 (PMC13308754; doi:10.2196/91396)
Supplement: Multimedia Appendix 1 [file rehab-v13-e91396-s001.docx]

# Supplementary Methods and Materials

## Knitting and post-processing

Each knitted prosthetic interface took approximately 2 hours to knit. After knitting, the prosthetic interface was mounted on a foam model of the user’s residual limb and heated to 150 °C then cooled to room temperature to stiffen the thermal-reactive yarns. The heating was done in an Ottobock 701E44=S infrared heating cabinet for 2 minutes. If the thermal-reactive yarn had not changed color after 2 minutes, the prototype was rotated so that the insufficiently heated areas face the heating elements at the top of the oven, and the prototype was heated again until the thermal-reactive yarn had darkened sufficiently to indicate adequate heating. After removing the interface from the oven, it was allowed to cool and stiffen on the foam model before removal.

## Rigid socket

After the knitted prosthetic interface was donned, a rigid socket connected to an artificial leg was placed over the knitted interface. A conventional lamination process was used to create the prosthetic socket. This involved the layering and bonding of fiberglass, nyglass, and perlyn materials over a positive plaster cast of the user’s residuum. Reinforcement materials, comprising unidirectional carbon fiber, were added along the LoNEs and over the distal lamination anchor. The layered mold was saturated with a liquid epoxy resin (Orthocryl Lamination Resin 80:20, Ottobock) and left to cure for 1 hour. After curing, the socket was marked for cutting by a qualified prosthetist (Author 1) in accordance with the LoNEs. The socket edges were polished with sandpaper and assembled to the prosthetic components (tube clamp adapter, pylon, Trias foot (Ottobock)) before being fitted and aligned to the user. The socket connected to the knitted prosthetic interface using an Ossur 621 pin lock system.

## Differential scanning calorimetry heating-cooling cycles

The steps for the differential scanning calorimetry heating and cooling cycles are listed in Table S1.

Table S1. Differential scanning calorimetry heating and cooling program.

| Step | Instruction | Duration or Rate |
| --- | --- | --- |
| 1 | Hold at 15.00 ^◦^C | 10.0 mins |
| 2 | Heat from 15.00 ^◦^C to 200.00 ^◦^C | 10.00 ^◦^C per min |
| 3 | Hold at 200.00 ^◦^C | 5.0 mins |
| 4 | Cool from 200.00 ^◦^C to 15.00 ^◦^C | 10.00 ^◦^C per min |
| 5 | Hold at 15.00 ^◦^C | 5.0 mins |
| 6 | Heat from 15.00 ^◦^C to 200.00 ^◦^C | 10.00 ^◦^C per min |
| 7 | Hold at 200.00 ^◦^C | 5.0 mins |
| 8 | Cool from 200.00 ^◦^C to 15.00 ^◦^C | 10.00 ^◦^C per min |
| 9 | Hold at 15.00 ^◦^C | 1.0 min |

## Uniaxial tensile test sample preparation

The tensile test samples were first knitted as tubes then fitted onto 180 x 180 x 15 mm plywood blocks to be heated in a Venticell Eco Line oven at 150 °C for 2 minutes, then flipped to heat the other side for another 2 minutes. After heating, the samples were removed from the wooden block, and the tube was cut along the sides to use the front and back faces for the mechanical tests. The center area was further cut to leave a 50 x 50 mm square to serve as the testing area, resulting in an I-shape sample. This method differs from the ISO standards to better allow the Instron machine’s jaws to grip the top and bottom segments, while keeping the testing area consistent across the samples. As the testing standard, ISO 13934-1, is intended for stiffer woven fabrics, the initial tested length of the knit samples was reduced to 50mm to accommodate the higher strain percentages that knitted fabrics can undergo so that it is within the physical constraints of the machine’s vertical testing length. Six samples were tested for each orientation and size combination, resulting in a total of 24 samples for each yarn material and stitch pattern combination.

## Vertical wicking rate test

For the vertical wicking rate test, to replicate the warm and humid conditions in Singapore, the samples were conditioned for at least 12 hours at 28.4 °C (the 24-hour mean temperature of May – the hottest month) and 83.5 % relative humidity (the mean annual relative humidity of Singapore) [39]. All tests were conducted in a humidity chamber (Cincinnati Sub-Zero Products, Inc., model: ZPHS-44-2-H/AC). The 170 x 40 mm samples were mounted vertically in a tray, then deionized water tinted with Bake King Concentrated Creme Bright Red food coloring was poured into the tray to the height of 20 mm above the base of the samples. Wicking time was defined as the time taken for the fluid to wick upwards to the 100 mm mark, and wicking rate was defined as wicking time divided by 80 mm (i.e., the distance between the 20 mm water level line and the 100 mm mark). Table S2 contains a summary of the different user test methods used.

Table S2. Summary of user test methods.

| Test | Description of Test |
| --- | --- |
| **Mobility** | |
| Range of Motion | Maximum extension and maximum flexion of knee, measured with a goniometer placed along the lateral midline of the thigh and the lateral midline of the lower leg. |
| 10-Minute Walk (10MWT) | Distance walked in a 10-minute duration while wearing the prosthesis. Skin temperature readings are also taken (see Thermal and Moisture Comfort). |
| 10-Meter Walk (10mWT) | Time taken to walk 10 meters while wearing the prosthesis. These readings were taken during the first 3 laps of the 10MWT. |
| Timed Up and Go (TUG) | Time taken to stand up from a chair, walk 3 meters, turn around, walk back to the chair, and sit down. |
| **Suspension** | Distal migration of the socket, measured using a static elongation test. |
| **Comfort** | |
| Mass | Mass of the total prosthetic system, the socket, and the interface. |
| Thermal and Moisture Comfort | Thermal comfort: Skin temperature at the muscle belly of the calf during the 10MWT. This test was performed twice, first with an amputee user in an indoor, air-conditioned environment, and second with a healthy user in an outdoor, sunny environment. The second user did a longer 15-minute walk.  Moisture comfort: User feedback and physical observation of the interfaces for moisture. |
| User Acceptance | User-reported outcomes using Socket Comfort Score, QUEST 2.0, unstructured interviews, and observations. |

### Range of motion

To measure the knee range of motion, the user sat on a chair with a fully extended knee. A goniometer (DMOrthotics Ltd.) was placed with one arm along the lateral midline of the thigh, aligning with the greater trochanter, and the other arm parallel to the lateral midline of the lower leg. Similarly, when measuring knee flexion, the goniometer was aligned with the same reference lines.

### Skin temperature measurements

The overall steps of the 10-Minute Walk Test (10MWT) with skin temperature measurement are outlined in Table S3. The steps were performed first with the current prosthesis (silicone liner). After a 1-hour rest period without the prosthesis and liner, the steps were repeated with the experimental prosthesis (knitted prosthetic interface with LoNE socket).

Table S3. Procedure for 10-Minute Walk Test with skin temperature measurement.

| Step | Instruction | Duration | Wearing |
| --- | --- | --- | --- |
| 1 | Rest | 30 mins | Nothing (bare skin) |
| 2 | *Wear interface* |  |  |
| 3 | Rest | 15 mins | Interface |
| 4 | *Remove interface for a short check of surface skin temperature* |  |  |
| 5 | *Wear interface and socket* |  |  |
| 6 | 10-Minute Walk Test | 10 mins | Interface and socket |
| 7 | *Remove interface and socket* |  |  |

When the test was repeated with a non-amputee user, for step 1, the user rested for only 5-15 minutes until the skin temperature reading stabilized. For step 6, the user walked for 15 minutes to ensure that sweat was generated.
